# Low Correlations between Intelligence and Big Five Personality Traits: Need to Broaden the Domain of Personality

**DOI:** 10.3390/jintelligence6020026

**Published:** 2018-05-01

**Authors:** Lazar Stankov

**Affiliations:** 1 School of Psychology, The University of Sydney, Sydney 2006, Australia; lazar.stankov@sydney.edu.au; 2 School of Psychology, The University of Southern Queensland, Toowoomba 4350, Australia

**Keywords:** intelligence, personality, Big Five, conservative syndrome, self-beliefs

## Abstract

The correlations between the measures of cognitive abilities and personality traits are known to be low. Our data based on the popular Big Five model of intelligence show that the highest correlations (up to r = 0.30) tend to occur with the Openness to Experience. Some recent developments in the studies of intelligence (e.g., emotional intelligence, complex problem solving and economic games) indicate that this link may become stronger in future. Furthermore, our studies of the processes in the “no-man’s-land” between intelligence and personality suggest that the non-cognitive constructs are correlated with both. These include the measures of social conservatism and self-beliefs. Importantly, the Big Five measures do not tap into either the dark traits associated with social conservatism or self-beliefs that are known to be good predictors of academic achievement. This paper argues that the personality domain should be broadened to include new constructs that have not been captured by the lexical approach employed in the development of the Big Five model. Furthermore, since the measures of confidence have the highest correlation with cognitive performance, we suggest that the trait of confidence may be a driver that leads to the separation of fluid and crystallized intelligence during development.

## 1. Background

The studies of personality and intelligence have a long history in psychology. However, the issues frequently raised in contemporary discussions about alternative ways of measuring individual differences and the need to strive for personality/intelligence integration can be traced back to the developments that took place midway through the 20th century. At the end of World War II and during the Cold War years, the multivariate statistical procedures became the main tool in the research on individual differences. At the same time, many new measures for assessing the non-clinical aspects of behavior in both domains were developed. Initially, it was not unusual to have the same researcher making important contributions to our knowledge on both personality and intelligence. The examples include the works of Guilford [1], Eysenck [2,3] and Cattell [4,5]. As expected, these researchers thought about the two domains in a similar way. Thus, even though personality tended to be assessed with self-report measures and intelligence was based on the measures requiring performance on cognitive tasks, essentially the same procedures of factor analysis were adopted and the same decision rules guiding the choice of solutions for factor interpretation were employed.

The development of tests of intelligence was originally bottom-up, with many tests of “higher mental processes” being created and tested. The theories of intelligence were based on the outcomes of factor analyses of large batteries of cognitive tests. The theory of fluid and crystallized intelligence, which has morphed into the CHC (Cattell-Horn-Carroll; [6]) model, can be seen as an attempt to organize a wide array of such tests into a hierarchical structure.

On the other hand, the measures of personality were developed utilizing two approaches. The lexical approach advocated by Cattell was similar in spirit to the work on intelligence as the idea was to use the lexicon to arrive at an exhaustive list of all personality-related terms. This led to the development of his 16PF questionnaire, which defined five higher-order factors [7]. The lexical approach was also employed as a map by Costa and McCrae [8] in the development of their version of the now popular Big Five model of personality although they did not use adjectives in the main body of their research. However, the advocates of this model did not study cognitive abilities and their approach to personality differed from that of the earlier investigators. For example, what Costa and McCrae [8] defined as facets are not the same as the lower-order factors in abilities or in the 16PF approach. The facets were generated by the top-down process, which involved accepting the existence of the Big Five and then constructing six scales for each to create a total of 30 facets. In other words, the facets are not akin to the ability tests or primary mental abilities, which were used as the building blocks in the studies of intelligence.

The second approach (such as Eysenck [3]) was sometimes referred to as “rational” in the sense that the items were based on the psychological analyses of behavior that often depended on clinical findings. From the very beginning, a criticism of the Big Five model was that it leaves out some of the important personality dimensions. One argument has been that a rational top-down approach can be used to fill in the missing parts. For example, it has been recently argued that the disintegration factor stands apart from the Big Five (Knežević et al. [9]). Studies of the disintegration factor included measures of both lexically derived and “rational” personality traits, which would be closer in spirit to what has evolved in the abilities domain.

## 2. Low Correlation between Intelligence and Big Five Measures

It is important to keep in mind that a large proportion of the work on personality assessments was carried out with no intention to link it to either intelligence or academic achievements. The traditional approach of individual differences was based on the assumption that the measures of personality and intelligence are uncorrelated (i.e., orthogonal) [10]. To illustrate this point, it is useful to consider the findings based on data from a cross-cultural study reported by Saucier et al. [11] that are presented in Table 1. This study employed a new measure of the Big Six (i.e., essentially the Big Five plus Honesty) described by Thalmayer and Saucier [12] and a Number Series test. The last row in Table 1 shows that Conscientiousness has a correlation of r = −0.02, while all other personality traits also have essentially zero correlations with the Number Series test that is a well-known measure of fluid intelligence. The highest correlation (r = 0.16) is with the Originality Big Six factor, which is akin to the Openness to Experience (or Intellect) in the Big Five model.

My aims in this paper are to: (a) examine whether recent developments in both personality and cognitive abilities hold promise for a rapprochement between these two domains; (b) summarize the findings from the two lines of enquiry, namely social conservatism and self-beliefs research, that have been studied in relationship to both personality and cognitive abilities; and (c) discuss the implications of the findings for future work on individual differences. In doing so, I will rely on the findings of our own team, which has been actively involved in studies of the individual differences in both cognitive abilities and personality for many years. Our findings have broader implications and there can be no doubt that other researchers may derive similar conclusions from their own work in similar or related areas.

## 3. Investment Theory and the Role of Openness to Experience

Some recent attempts to link cognitive abilities and personality were inspired by Cattell’s [5] investment theory. According to this theory, crystallized intelligence (Gf) emerges from fluid (Gf) abilities depending on motivation, interest and effort invested in pursuing particular activities in life. The correlations between personality and cognitive abilities are used to identify non-cognitive processes that may drive the investment of effort. Ackerman [14] pointed out that aspects of personality (Openness to Experience) and interests (e.g., Realistic, Investigative and Artistic) are critical for the development of crystallized intelligence. Indeed, among the Big Five (to be referred to as Big Five/Six in the remainder of this paper), the measures of Openness to Experience have been reported to have the highest (but weak to moderate) correlation with intelligence. For example, von Stumm and Ackerman [15] summarized the personality traits that influence when, where and how people invest their time and effort into learning. Their meta-analysis reported a correlation of r = 0.30 between investment traits, such as Openness to Experience, and cognitive abilities (see also Ziegler et al. [16]). Our own work has been broadly consistent with these findings as most of our correlations between intelligence and Openness to Experience have been in the range of r = 0.20−0.30 [17].

Following on from the meta-analysis of the relationship between the Big Five and academic performance, Poropat [18] showed that Conscientiousness was linked to measures of achievement. This link was not explicitly related to the investment theory. He reported that Conscientiousness is a significant but slightly worse predictor of academic performance compared to intelligence itself, particularly among groups with a restricted range, such as samples composed of University students. The investment theory can be linked to this finding if we assume that academic performance reflects the processes of crystallized intelligence. However, Conscientiousness tends to have a much lower correlation with intelligence than Openness to Experience in our own studies^1^. It is often lower than the sample weighted r of 0.19, which was reported by Poropat [18] as the estimated correlation between Conscientiousness and academic achievement^1^.

In our work, the correlations above r = 0.20 are treated as noteworthy since this indicates that only about 4% of the variance is in common between the correlated measures. This means that among the Big Five measures of personality, Openness to Experience provides the best account of the processes implied by the investment theory. Are there other personality traits that are poorly assessed by the Big Five model but have equal or higher correlations with cognitive abilities?

## 4. Recent Developments in Studies of Individual Differences

Overall, the cognitive abilities and personality as measured by the now popular Big Five model are largely uncorrelated and similar to what has been the case in the past (see Colis and Messick, [19]). However, the field of individual differences is developing and closer interactions between the domains may be identified in the future.

Three issues need to be highlighted. First, there are suggestions that emphasis on the general factor of intelligence may have had a detrimental effect on the search for individual differences in cognitive processes outside of the traditional areas covered by the label of “higher mental processes” (see Stankov [20]). New cognitive tests for the previously neglected domains are currently being explored. Second, as pointed out above, the Big Five/Six theory does not cover the personality domain extensively enough and there are likely to be additional personality factors, some of which may be correlated with cognitive abilities. Third, many measures of individual differences are currently not captured either by the Big Five/Six measures of personality or by the typical tests of intelligence. They can be thought of as being located on the “no-man’s-land” between the two domains [21].

In this paper, some of the new developments in the assessment of individual differences will be briefly described. It is possible that they will shed a different light on the personality−intelligence relationship.

### 4.1. Recent Expansions of the Cognitive Domain and Potential Links to Personality

Stankov [20] pointed out that several areas of cognitive functioning have been poorly explored in studies of individual differences. Some of the neglected areas, such as the measures related to sensory processing, are unlikely to be related to personality traits, but others may. The most obvious is the ability measure of emotional intelligence, which does seem to fit into the CHC model but still has an unusually high (r = 0.42) correlation with the Big Five measure of Agreeableness. This correlation may be due to the scoring method employed rather than to a substantive relationship between the domains. That issue will need to be addressed in future empirical studies.

Another area of potential interest is the study of complex problem-solving skills. These skills are assessed by asking participants to imagine that they are, say, a mayor of a small town and have to engage in a series of decisions related to this role. Although there is enough evidence that measures of this type correlate with the traditional tests of intelligence, Stankov [20] pointed out that the correlations between different tests of complex problem-solving skills are relatively low and they are unlikely to define a separate factor. Given that the real-life feel of these types of assessments is stronger than that of the IQ tests, it can be expected that personality traits may interact with cognitive abilities in the performance on at least some complex problem-solving tasks.

The links between cognitive abilities, personality and economic games are possibly less obvious. In general, typical economic games are not designed to assess intelligence. They tend to examine behavior, such as generosity, egalitarianism and reciprocity, and in studying these, they focus on pro-social personality traits, such as agreeableness, honesty–humility and extraversion (see Zhao, Ferguson and Smillie [22]), and economic preferences (see Almlund et al. [23]). However, it would be useful to develop economic games that emphasize both the cognitive processes and personality traits and to look at their interactions in shaping behavior. It is also conceivable that as discussed by Stanovich [24], rationality has both cognitive and personality components.

### 4.2. Recent Expansions of the Domain of Personality Do Not Change Its Relationship to Cognitive Abilities

Two areas that have intuitive links to personality have been the subject of a noticeable increase in research activity over the past couple of decades. One of these is the area of *positive psychology*, which emphasizes the role of personal qualities and how they contribute to subjective well-being. The development of instruments to measure happiness, life satisfaction and the related remedial area of mindfulness was possibly the most important contribution to this line of research. Well-being itself is usually not considered a personality trait as it is typically treated as an outcome variable. The attempts to relate the Big Five factors to the measures of happiness and life satisfaction occasionally point to the predictive role of extraversion. However, our own work suggests that a well-being measure loads on the Life Satisfaction factor together with four Big Five scales and therefore, points to the involvement of the General Factor of Personality (GFP; [25]) rather than any particular personality trait. The same well-being measure, together with Emotional Stability (the opposite of Neuroticism), also has negative loadings on the Depression factor [26]. The correlations between intelligence and measures of well-being tend to be low.

The second area arises from the emphasis on “dark triads” and related *negative psychology* traits. The list of dark triads originally included psychopathy, Machiavellianism and narcissism, while sadism was added subsequently. A recent meta-analysis of the relationship between dark triads and Big Five/Six measures by Muris et al. [27] concluded that the dark triad traits are substantially intercorrelated and related to the Big Five/Six personality factors of agreeableness and the HEXACO factor of honesty−humility. These authors questioned whether the dark triad traits are sufficiently distinct and argue that the method of currently measuring them is too simple to capture the malevolent sides of personality ([27], p. 183). As we shall see shortly, some aspects of social conservatism and the previously mentioned disintegration factor may also turn out to be related to the dark traits of negative psychology. Again, there is currently no evidence that either dark triads or disintegration correlate with measures of cognitive abilities.

Overall, it appears that even though both domains of cognitive abilities and personality have been expanding, it is mostly the new work in the area of cognitive abilities that promises to open a link to personality. The new developments in the domain of personality are not oriented towards constructing measures that would expand the link to cognitive abilities. Instead, there appears to be a preference for maintaining the status quo exhibited in the Big Five/Six model as it relates to well-being and malevolent behavior.

### 4.3. No-Man’s-Land between Personality and Cognitive Abilities

It is becoming increasingly clear that some aspects of behavior are not adequately captured either by the existing Big Five measures of personality or by cognitive abilities. For example, contemporary sport and military psychology as well as the psychological aspects of social media call for new assessment procedures. To address this issue, many new tests and scales have been constructed but their relationships to the existing measures have often not been properly examined. Some of these new measures may be considered to be an aspect of personality, some may capture aspects of cognitive ability, while others focus on neither or both at the same time.

In this section, I consider the two areas that have been related to cognitive abilities and personality, which are namely social conservatism and non-cognitive measures used in educational assessment. Social conservatism is defined mostly in terms of social attitudes and is conceptually closer to personality, while the educational measures are more closely related to cognitive abilities.

#### 4.3.1. Big Five/Six and Cognitive Ability Are Weak Predictors of Conservative Syndrome and Militant Extremist Mindset (MEM)

Personality traits are sometimes confused with social attitudes. This latter term is usually applied to authoritarianism, dogmatism and related constructs. In the past, Machiavellianism was classified as a social attitude, but the contemporary inclination is to treat it as one component of the dark triads, which are also sometimes interpreted as aspects of personality. This points to a blurred boundary between personality and social attitudes.

Although both personality traits and social attitudes are dispositions (i.e., latent constructs inferred from verbal and non-verbal measures), there are differences between them. A personality trait is a characteristic of an individual that influences a range of her/his verbal or non-verbal behavior. These traits are not evaluative in nature. Social attitude is a person’s disposition to evaluate, which involves responding favorably/unfavorably (e.g., pleasant or unpleasant) to a person, object, event or institution. It is also assumed that social attitudes involve three component processes: cognitive (e.g., beliefs about an object); affective (feelings towards an object, such as disgust); and conative (i.e., behavioral inclinations and intentions to act towards an object). This suggests that they may indeed be placed somewhere between personality and cognitive ability traits.

Stankov and Lee [13] analyzed 19 putative measures of social attitudes and identified three factors: *Nastiness* (“characterized by acceptance of the use of violence to resolve social problems and the use of dubious means to achieve selfish and materialistic goals which, in turn, are seen as being most important”, p. 6); *Religiosity*; and *Morality* (“the endorsement and appreciation of the values and moral principles that facilitate communal life, interactions with fellow human beings and encouragement of selfless behavior”, p. 6). At the country level of analysis, these three factors define a single Conservatism/Liberalism dimension. As the correlation between Nastiness and Morality is low at the individual level of analysis, a second-order Conservatism/Liberalism factor is weak and we refer to the common social attitude disposition as the Conservative Syndrome.

For those interested in cross-cultural comparisons, Morality is the least important dimension. This is because we found that the differences between countries are very small and scores on this factor tend to have virtually zero correlations with many country-level political and economic indices of development. However, the individual differences in the measures of morality are pronounced to a similar extent as Nastiness and Religiosity. As seen in Table 1, Morality has a virtually zero (r = −0.01) correlation with the Number Series test. As expected, the personality scales of Conscientiousness (r = 0.27) and Honesty (r = 0.24) have relatively weak correlations with Morality. The other four correlations of Morality with the Big Five/Six personality traits are low.

The most important Conservative Syndrome factors are Nastiness and Religiosity. With respect to Nastiness, it is useful to note that as expected, all its correlations with Big Five/Six personality scales are negative (see the first row in Table 1). The highest correlations are still low to moderate with Honesty (r = −0.32) and Originality (r = −0.30), followed by the weak correlation with Extraversion (r = −0.24). Its correlation with the Number Series test (r = −0.17) is below our threshold for being noteworthy. Stankov [28] also reported that Honesty has a low negative loading on a broad Nastiness/Social Dominance factor.

As seen in Table 1, Religiosity is correlated with the Number Series test at the borderline level (r = −0.20) and has slightly higher correlations with Originality (r = −0.22) and Conscientiousness (r = 0.23). Thus, people that have a high Religiosity score tend to be somewhat less able to solve fluid intelligence tasks and are slightly more conscientious and closed-minded. These are not high correlations, as can also be ascertained from the low loading of Conscientiousness on a broad Religiosity factor in Stankov [28]. It should be mentioned that there is extensive literature on the relationship between cognitive abilities and religiosity. A meta-analysis reported by Zuckerman, Silberman and Hall [29] was based on 63 empirical studies from 52 sources, while several recent papers, including a Special Issue of the journal *Intelligence*, have been devoted to the same topic. According to Zuckerman et al. [29], the correlations between intelligence and strength of religious beliefs at the individual level range from r = −0.20 to r = −0.25. Our own finding is clearly at the lower end of this range.

It is also important to point out that both Nastiness and Religiosity are aspects of the contemporary militant extremist mindset (MEM; [30]). Thus, persons that have a high MEM score may also score somewhat lower on honesty, originality and cognitive ability. However, a recent study by Medjedovic and Knezevic [31] reported that the Big Five/Six measures of personality do not predict MEM at all once the correlations with what they refer to as dark and psychotic-like traits are taken into account. In their study, Nastiness (Pro-violence) has significant moderate correlations with dark measures of sadism and callous affect, while Religiosity correlates with the new personality factor of disintegration.

It is apparent that the effect sizes for predicting both Nastiness and Religiosity from the Big Five/Six personality and cognitive ability measures are small. Nevertheless, the findings appear to be replicable and they do make psychological sense. However, low−moderate correlations indicate that social conservatism stands apart from these two domains of individual differences. In turn, both social conservatism and MEM are related to the dark dispositions, some of which may be classified as rightfully belonging to the domain of personality but are not captured by the Big Five/Six model.

#### 4.3.2. Self-Beliefs Are Better Predictors of Cognitive Performance than Big Five Measures of Personality

Given that personality and cognitive abilities have low correlations among themselves, educational psychologists have explored the role of a large number of non-cognitive measures that may predict achievement. Some of these are sociological or demographic, while others are student-, teacher-, parent- or system-related. A considerable number were psychological in nature, including the academic interests of students, the use of learning strategies, motivation (intrinsic/extrinsic), goal structure and so on. Most of these measures have correlations with achievement scores that are less than r = 0.20. For example, the achievement in mathematics had a correlation of r = 0.11 with the measures of perseverance and r = 0.17 with openness for problem solving [32].

Among the non-cognitive measures that had noteworthy (i.e., higher than r = 0.20) correlations with the Programme for International Student Assessment (PISA) mathematics achievement scores, three constructs stand out:

- *Mathematics self-efficacy*. Students were asked to rate their confidence in their ability to solve each of seven hypothetical mathematic problems but they were not asked to carry out the necessary calculations. An example item is: “How confident do you feel about … calculating the number of square meters of tile you need to cover a floor”.
- *Mathematics self-concept*. Five items taken from PISA 2003 were supplemented with three additional items. An example item is: “In my mathematics class, I understand even the most difficult work”.
- *Mathematics anxiety.* Five items from PISA 2003 were supplemented with 14 additional statements. All items were negatively worded. An example item: “I get very nervous doing mathematics problems”.

Table 2 presents their correlations with PISA mathematics achievement scores. Although the correlations with self-concept are noteworthy but weak, both self-efficacy and anxiety have significant moderate correlations with cognitive ability. Mathematics self-efficacy and mathematics anxiety have higher correlations with mathematics achievement compared to any Big Five/Six measure of personality. Collectively, these “self-“measures are referred to as self-beliefs. In the educational literature, they are sometimes discussed in relation to resiliency, grit, mental toughness and so on. A fourth measure of self-belief was not employed in PISA surveys but our own work indicates that this is the best non-cognitive predictor of academic achievement:

- *Confidence.* Following each mathematics item attempted in the achievement test, students were asked to rate on an 11-point scale (0% to 100%) their confidence that the answer they gave was correct.

In general, confidence has a correlation of r = 0.40−0.60 with cognitive ability and self-efficacy measures. Stankov, Morony and Lee [33] found that the above four non-cognitive measures define a separate factor of self-beliefs and Stankov, Lee, Luo and Hogan [34] reported that confidence and anxiety are the best predictors of academic achievement in both mathematics and English.

As gathered from the labels for the four constructs of self-beliefs, the first three are domain-specific as they refer to the particular academic area of mathematics. It is well known that the mathematics self-concept is negatively (or zero) correlated with verbal (e.g., English) self-concept and the same holds for self-efficacy. Both may be seen as the reflections of self-esteem. Although mathematics anxiety on its own is domain-specific, we shall see shortly that it should be treated as a low-level marker of test-related and general anxiety.

#### 4.3.3. Confidence: Personality Trait, Domain-Specific Metacognitive Process, or …?

The status of confidence needs to be clarified. Its origin is in the area of decision-making and when used in psychological testing, it is often linked to the metacognitive process of self-monitoring, which is essentially how well we know that we have solved each of a series of test items correctly. It is similar to the PISA’s measure of self-efficacy in the sense that it refers to a particular cognitive act and consequently, some psychologists do not accept the interpretation of confidence as a broad personality trait. This is fine, of course. However, it needs to be kept in mind that we have repeatedly found a broad confidence factor. For example, Kleitman and Stankov [35] reported that confidence ratings from five tests of intelligence define a confidence factor that is clearly separate from the ability factor based on the accuracy scores calculated from the same five tests. This may be interpreted as suggesting that confidence may be a general trait, which is akin to the ‘g’ factor of intelligence (see also Stankov [36]).

To illustrate this point, it is useful to consider the results reported in Table 3, which have been adapted from Stankov et al. ([34], p. 572). This table presents the findings from four tests of mathematics achievement (Number, Algebra, Geometry and Statistics) that were given to High School students. From each test, two scores were calculated: Accuracy (a typical achievement score) and Confidence. As seen from the comparison of arithmetic means, the confidence mean is higher than the accuracy mean for every mathematics task, demonstrating a typical bias towards overconfidence. The outcomes of the exploratory factor analysis were the two factors of Accuracy and Confidence that were extracted from the above correlational matrix and are in complete agreement with the findings summarized above [34]. Although the factor loadings represent the summary of the relationships, it may be useful to call the readers’ attention to the correlational matrix itself.

Three points are worth noting. First, the correlations between the four confidence scores presented in the lower right-hand corner of Table 3 are much higher than the correlations among the accuracy scores in the top left-hand section. Thus, the value of the common variance among the four measures of mathematics achievement is smaller than the common variance of confidence scores from the same tests. Second, the correlations between accuracy and confidence from the same test have a range of r = 0.40−0.67. This is similar to the typical correlations between fluid and crystallized intelligence, suggesting that the accuracy and confidence measures capture sufficiently different processes. Third, in the presence of domain specificity, we can expect that the correlations between Accuracy and Confidence from the same test will be high and the correlations of the same measures from different tests will be small. In other words, the 4 × 4 lower-left corner sub-matrix in Table 3 will have high diagonal (bold font) and low off-diagonal values. As can be seen, the diagonal values for Number and Algebra (r = 0.65 and r = 0.67, respectively) are indeed higher than off-diagonal values, but the same is not true for Geometry and Statistics. Thus, the correlation between the Confidence score for Algebra and Accuracy score for Geometry (r = 0.44) is higher than the correlation between the Accuracy and Confidence scores from Geometry itself (r = 0.40). We can conclude that the evidence to date indicates that Confidence is not domain-specific and in our experience it can be reliably assessed using any type of cognitive test.

#### 4.3.4. Revisiting the Investment Theory: Crucial Role of Confidence?

If we accept the argument that the best candidate for the force driving the transition from fluid to crystallized intelligence in Cattell’s investment theory is a non-cognitive psychological process that has the highest correlation with measures of cognitive abilities, it can be claimed that confidence, not Openness to Experience, plays the most important role. Thus, people who are good and confident in judging the quality of their performance after the completion of a task (i.e., test item) and are consistent in doing it well over many items in a test may be seen as being effective in self-monitoring. This metacognitive awareness of the quality of their work can facilitate a person’s engagement in activities related to their interests, whatever these are, and lead to an investment of effort for the purpose of mastery. In other words, confidence in one’s decision-making may free a person to choose whatever she/he fancies in life.

There are important educational implications that stem from the likely existence of a broad confidence factor and the well-known fact that during childhood development, fluid and crystallized intelligence gradually separate into distinct factors and become clearly recognizable only in adolescence. From this perspective, the existence of domain-specific self-beliefs should be seen as a hindrance that needs to be removed. The idea that a person is “good” at mathematics and therefore, “bad” in language-related skills is something that must come from the outside (e.g., contextual and peer influences) since a general factor of intelligence and broad (or general) factor of confidence argues strongly against domain specificity. Educators should point to the existence and importance of domain generality. Essentially, if you are good at one thing, you are likely to be good at everything else you choose to be involved in.

#### 4.3.5. Apart from Math Anxiety, Self-Beliefs Have Low Correlations with the Big Five

It is logical to ask whether the non-cognitive measures of self-beliefs have noteworthy correlations with the Big Five/Six personality traits. Unfortunately, relevant information is not available from the PISA surveys, since these did not include measures of personality. However, Stajkovic et al. [37] reported that *self-efficacy* has noteworthy but relatively low correlations with Conscientiousness (r = 0.25) and Emotional Stability (r = 0.22) and close to zero correlations with all other Big Five factors. Although *self-concept* was used to predict academic achievement together with the Big Five personality factors, there seems to be no explicit information about the correlations of self-concepts with personality in published studies. Dowker, Sarkar and Looi [38] reported that the correlation between mathematics *anxiety* and test anxiety is in the range of r = 0.30−0.50 and that both are related to general anxiety and the neuroticism factor. Kleitman and Costa [39] reported that *confidence* has a correlation of r = 0.20 only with the Intellect Big Five factor. All other Big Five personality factors have correlations with confidence that are close to zero. Thus, apart from the measure of mathematics anxiety, the correlations of self-beliefs with the Big Five measures of personality tend to be low.

## 5. Need to Broaden the Personality Domain beyond the Big Five Model

The correlations between the measures of cognitive abilities and the Big Five/Six scales that are the most popular measures of personality remain low. Perhaps some will argue that the situation should stay as it is, since the zero correlation allows for an easy choice of candidate variables that may have incremental validity over and above cognitive abilities. I have difficulty in agreeing with this argument since in my experience, the measures of Big Five personality rarely have correlations that are higher than r = 0.30 with any criterion variable and therefore, are unlikely to add much predictive value. The origin of the Big Five is in the military environment and Conscientiousness may be of some relevance for business. Its relevance for predicting academic achievement is limited. Walter Mischel [40] made a similar point half a century ago when he opened the person−situation debate. The ensuing discussions did not lead to the abandonment of trait psychology and, to be fair, this was due in part to the emergence and popularity of the Big Five model. However, I believe that limiting the domain of personality to the Big Five/Six model is too restrictive. Broadening the domain of personality to encompass psychological processes outside this model may lead to improved predictive validity of the personality measures, which will most likely increase correlations with cognitive abilities.

The heyday of the lexical approach and the Big Five/Six model should be over. It will be useful to reopen debate about the definition of personality and delineate the constructs that may qualify as such. Some of these constructs may come from close theoretical areas, such as social psychology, or possibly from practical fields, such as educational or political psychology. The strategy may involve the use of the Big Five/Six scales. Their relationship to the measures chosen on the basis of “rational” analysis should be studied empirically.

Another possibility is to elevate some of the facets of the Big Five traits. For example, DeYoung [41] proposed a model of the Openness/Intellect that claims that Openness and Intellect measure different aspects of personality and encompass six facets (intelligence, intellectual engagement, innovation/imagination, aestheticism, fantasy/absorption and apophenia). One may ask if it makes sense to place intelligence within this model and more broadly, whether it is reasonable to treat Openness and Intellect as different traits.

Based on our work, the candidate non-lexical measures from the “darkness” region may include psychopathy and disintegration. For example, an important component of disintegration is apophenia, which is a tendency to see patterns in randomness (Knezevic et al. [9]). Further work may also uncover the relationship of cognitive abilities with narcissism, sadism and aspects of religiosity, morality, nastiness and conservatism. From the self-beliefs constructs we have looked at, confidence and anxiety are the obvious candidates. Self-esteem, which is assumed to underlie several constructs of self-beliefs, may be added to the list. Given that well-being, happiness and life satisfaction cannot be accounted for by economic measures on their own, it is conceivable that these may reflect a personality trait at a higher-order level. However, this list is again based on our own experiences and other researchers are likely to add many, many more constructs.

## 6. Summary and Discussion

The correlations between personality captured by the Big Five/Six model and cognitive abilities tend to be low. Given that many activities in real life appear to be related to both sources of individual differences, it can be expected that at least some personality measures will have noteworthy correlations with cognitive performance. The currently popular theory of intelligence, the Cattell-Horn-Carroll (CHC; [6]) model, also postulates that the separation between fluid and crystallized intelligence during development is related to non-cognitive processes. This paper summarizes our own findings related to low correlations and reviews some recent studies on cognitive ability and personality with an aim to achieve closer interactions between the two fields in future. It also reviews the findings from the studies of Conservative Syndrome and militant extremist mindset (MEM) as well as from the studies of self-beliefs.

New developments in the area of cognitive abilities, such as emotional intelligence and complex problem solving, hold promise of being related to personality measures. New developments in personality research are less promising in their potential correlation with abilities. Conservative Syndrome and MEM both have correlations of approximately r = 0.30 and lower with the Big Five/Six measures and with cognitive abilities. However, self-beliefs are highly correlated with cognitive abilities and have low correlations with the Big Five/Six measures.

It is well known that cognitive abilities are better predictors of academic and job performance compared to typical measures of personality. It follows that changes in our conceptualization of personality hold greater promise as a way of achieving improved personality−ability integration. My argument is that the domain of personality is broader than what has been identified through the lexical approach, with the Big Five/Six model having hijacked the term of “personality”. Based on our own findings, personality may be expanded to include some “dark” traits and self-beliefs, but other researchers may have further suggestions. Among the self-beliefs, the measures of confidence are particularly promising, which can be seen both as a likely driving force within Cattell’s [5] investment theory and as an assessment that may lead to an improvement in predictive validity over and above cognitive abilities.

## Figures and Tables

**Table 1:** Correlations between Social Attitudes, Personality and Number Series Test (N = 8883).

	Big Six Personality Traits [12]						Number Series Test
	Conscientiousness	Honesty	Agreeableness	Resilience	Extraversion	Originality	
Nastiness	−0.18	−0.32	−0.21	−0.11	−0.24	−0.30	−0.17
Religiosity	0.23	0.14	−0.01	−0.00	−0.07	−0.22	−0.20
Morality	0.27	0.24	−0.10	−0.04	0.15	0.09	−0.01
Number Series	−0.02	0.02	0.04	0.07	0.07	0.16	

Note: The correlations between the Big Six and social attitudes (Nastiness, Religiosity and Morality) are adapted from Stankov ([13] Table 2, p. 142).

**Table 2:** The correlations between the PISA Mathematics Achievement Scores and Self-beliefs (N = 485,490 for PISA 2012; N = 276,165 for PISA 2003) ^1^.

	Variable Label	2012	2003
1	Mathematics self-efficacy	0.461	0.458
2	Mathematics self-concept	0.264	0.275
3	Mathematics anxiety	−0.365	−0.378

^1^ From Lee and Stankov [32].

**Table 3:** Mathematics achievement (accuracy) and confidence correlations (N = 1727).

		Accuracy				Confidence			
		Number	Algebra	Geometry	Statistics	Number	Algebra	Geometry	Statistics
Accuracy	Number	1.00							
	Algebra	0.60	1.00						
	Geometry	0.42	0.44	1.00					
	Statistics	0.47	0.50	0.39	1.00				
Confidence	Number	**0.65**	0.55	0.39	0.43	1.00			
	Algebra	0.60	**0.67**	0.44	0.48	0.83	1.00		
	Geometry	0.38	0.40	**0.40**	0.34	0.67	0.75	1.00	
	Statistics	0.49	0.48	0.38	**0.50**	0.72	0.77	0.78	1.00
Means		60.08	51.06	40.46	48.73	64.05	54.59	42.40	52.18
Standard Deviations		25.77	25.01	20.86	27.89	27.07	30.57	27.08	29.74

Note: Correlations between accuracy and confidence scores from the same test are in bold font.

## Footnotes

1 Our own findings were not included in meta-analyses of the relationship between cognitive abilities and personality.
